# Supplementary material for: How emotional changes affect skin odor and its impact on others
Source: PLoS One. 2022 Jun 30;17(6):e0270457. doi: 10.1371/journal.pone.0270457 (PMC9246182; doi:10.1371/journal.pone.0270457)
Supplement: Attachment — Submitted filename: Response to Reviewer2(M_Katsuyama).docx [file pone.0270457.s002.docx]

Thank you very much for your detailed comments. I have italicized your comments and bolded my responses.

*Reviewer #2: This study investigates relations between human subjective responses obtained by POMS and physiological responses such as gas evaporated from human skin surface of non-dominant hand, cortisol in saliva, and heart rate under a stressful state. In addition, it aims to create an odour model under stress/tense condition with which those who smell the odour are emotionally affected.
As a whole, the results in this study are shown in appropriate ways and are written in a comprehensive manner, suggesting that the main objective physiological response, skin gas, reflects subjective responses and correlates with other physiological indices reflecting sympathetic nervous system activity. Ethical consideration throughout the experiment is also appropriate. Sample size, experimental design, and analysis are properly administered as a whole. Thus this manuscript can be accepted after certain corrections and adding further explanations required to clarify the experimental procedure and its limitations. At the same time, the manuscript somewhat lacks balance of information volume in a way that some information on the experimental procedure is sufficient (such as the method to collect skin gas) and others insufficient. Especially descriptions on behavioural procedure is insufficient.
The following information includes the points that require further corrections or explanations are necessary.*

**Thank you for your comments. I apologize for the lack of organization in the text. I will provide an overall organized explanation before responding to each comment. We have also reorganized the abstract. First, the expression "the odor similar to sulfur-containing compounds " is too broad in meaning, so we changed the characteristic smell we found to a more specific expression: "the characteristic stir-fried leek-like odor".**

**The study consists of three points.**

**#1 The discovery of an odor like that of stir-fried leeks from the non-dominant hand of subjects in a state of stress, as supported by physiological indices**

**#2 Identification of the components of the characteristic leek odor**

**#3 By smelling the identified components, even in non-stressful situations, one can subjectively perceive tension and fatigue. In other words, it was shown that feelings can be propagated by smell.**

**Below, I have provided answers and responses to some of the points you raised.**

*Validity of the use of the term “psychological-” should be considered. The term has long been widely and inappropriately used in a wide variety of research papers to only represent human subjective responses, whereas psychology includes both subjective and objective aspects. Considering that various responses observed in animal psychology are typically non-verbal and thus must be measured only in objective manners, researchers no longer should not refer to psychological responses only as subjective. Furthermore, this study only employed POMS to evaluated participants’ subjective responses. The authors should alter the use of the term “psychological” into “subjective”, or at least define how the term is chosen to represent POMS data.*

**<Response :>**

**I understand your point and have changed 'psychological' to 'subjective'.**

*l-232*
*So called prescribed properties in this study should be described delicately since subjective measures should have been obtained with a more sufficient care. For example, the participants are set in the interview situation where they feel certain stress (l-232). Thus it is natural that they evaluate their subjective state in a emotionally-negative manner expecting what the experimenters anticipate. Thus how the properties are put into consideration in this study should at least be mentioned.*

**<Response>**

**The interview was, as you stated, a situation where we expected the participants to feel stressed. We showed that the interview produced a stress state, as we had expected, by checking fluctuations in the physiological indicators via electrocardiographic activity and salivary cortisol levels.**

*l-119*

*Furthermore, the way how the experts evaluated the skin gas seems inappropriate in a sense the odors are evaluated without a blind method (l-119). If done in a blind manner, it should be noted, too.*
**<Response>**

**The skin gas samples were numbered and the assessors were not informed of the background of the samples to help ensure a blind evaluation. The raters shared the perception of a distinctive odor like that of stir-fried leeks and rated its intensity on a five-point scale from “not smelling at all” to “strongly smelling”, according to their respective evaluation criteria. I have added the operating instructions.**

*Experimental design where the variable “stressful condition” is manipulated is not appropriate and this should also be mentioned in the manuscript. Admitting that the stressful condition is fairly controlled in a way the dependent variables are properly collected, the interview condition could have been more rigorously controlled. To be specific, a valid setting would be setting stressful (experimental condition) and non-stressful (control condition) interview/conversation conditions so that various activity levels are closer enough to each other to compare the difference in a more controlled way(l-251). Biking exercise is quantitatively and qualitatively different as an activity that it can not be rigorously compared. In that sense, cortisol level in saliva and heart rate are not compared in an ideal manner in this study. Such limitation of this study should at least be noted.*
**<Response>**

**Thank you very much for the advice. In this exam, we created an environment where subjects were interviewed by people they did not know as a means of creating stress. Regardless of the contents of the interview, talking with a stranger can be stressful. For the relaxed state, we asked the subjects to read magazines that didn’t contain any stimulating topics.**

**We needed to prove that the characteristic leek-like odor produced during the interview was not simply due to increased sweating. Therefore, we had subjects exercise on a stationary bike as a means of increasing their heart rate and perspiration without increasing stress levels, and checked the odor of skin gases collected during this exercise.**

*l-112
Time schedule of the experimental procudure as a whole should be illustrated in a more comprehensive way such as with a timeline figure. It is also unclear if the participants in each experiment are partly the same or different, which should also be noted.*
**<Response>**

**A time schedule for the entire skin gas sampling experiment process during tension-induced interviews has been added.**

**Participants in the verification of the effect for subjective reaction of the model tension odor were some of the participants in the interview test and were asked to cooperate on another day.**

*l-113
Cortisol level can also be affected not only by the time of the day but also by the experimental surroundings which should easily affect sympathetic nervous system. Thus, the samples should have been collected on a different day at the same laboratory where the experiment was conducted. In addition, visual analogue scale (VAS) is recently used widely to evaluate various subjective responses in researches including studies on olfactory function. Furthermore, a Likert-style scale has fatal problems including anchoring effect that evaluators’s memory on the previous score affects following evaluations if done more than once in one experiment. It is partly because of the the problem that it can only be treated as a nominal or ordinal scale. Although such scales has long be used especially in the psychology field, the authors should at least explain disadvantages of such scales considering that there are accumulating numbers of studies claiming such questions.*
**<Response>**

**I understand your point about the timing of the saliva collection.**

**In this experiment, although the saliva from after the subjects read was collected during the same time of day the saliva was collected at home, the results were also included in the graph (fig. 4).In addition, I understand that the POMS Likert scale references the previous score, which causes an anchoring effect on the next evaluation. In the main text, we have added an explanation of its disadvantages.**

*l-193
The participants in this study are biased in age and gender. The authors should explain backgrounds of the distribution.*

**<Response>**

**As the skin gas sampling in this study was performed on the hands, it was necessary to avoid tobacco odor contaminants found on the hands of smokers, so the target population was non-smokers. In addition, as body odor is known to differ between men and women and with age, the study was initially restricted to women. I have added an explanation about this to the text.**

*l-206*
*There are various subjective evaluation scales other than POMS. Although POMS is certainly one of the best measures and the authors properly explain the questionnaire in detail, the factors to be employed as compared with other famous measures should be simply noted. Here again, how the participants evaluated the scale (not VAS?) should be mentioned since they evaluated their state twice in the experiment.*

**<Response>**

**As you pointed out, there are various subjective evaluation scales. POMS was selected because the subjects of this study were healthy people and POMS has the ability to cover seven subjective emotional states. Subjects evaluate themselves twice in one experiment on a 5-point Likert scale. We described the disadvantages of answering using the Likert scale within the same study.**
